# SARS-CoV-2 Omicron variant is highly sensitive to molnupiravir, nirmatrelvir, and the combination

**DOI:** 10.1038/s41422-022-00618-w

**Published:** 2022-01-20

**Authors:** Pengfei Li, Yining Wang, Marla Lavrijsen, Mart M. Lamers, Annemarie C. de Vries, Robbert J. Rottier, Marco J. Bruno, Maikel P. Peppelenbosch, Bart L. Haagmans, Qiuwei Pan

**Affiliations:** 1 Department of Gastroenterology and Hepatology, Erasmus MC-University Medical Center, Rotterdam, the Netherlands; 2 Viroscience Department, Erasmus MC-University Medical Center, Rotterdam, the Netherlands; 3 Department of Pediatric Surgery, Erasmus MC-Sophia Children’s Hospital, Rotterdam, the Netherlands; 4 Department of Cell Biology, Erasmus MC-University Medical Center, Rotterdam, the Netherlands

**Keywords:** Molecular biology, Immunology

Dear Editor,

Since the first outbreak in late 2019, the RNA genome of SARS-CoV-2 has been undergoing constant evolution. This is largely attributed to the viral polymerase that is intrinsically error prone and the selection pressures exerted by the host immune system. Several variants of concern harboring multiple mutations in the spike protein have emerged in the past year. The currently fast-spreading Omicron variant contains many more mutations compared with the previous variants, and most of these mutations are located around the receptor binding domain of the spike protein.^1^ This would dramatically, although not completely, compromise the efficacy of the existing COVID-19 vaccines.^2^ Nearly all the monoclonal antibodies developed for treating COVID-19 are directed to the spike protein to prevent virus entry into human cells. Thus, it is not surprising that most of these antibodies are not effective against the Omicron variant.^3^ Prompted by the potentially devastating impact of this new variant, we evaluated the effectiveness of two clinically available direct-acting antiviral agents on the Omicron variant in relevant experimental models.

Molnupiravir is a small-molecule prodrug of the nucleoside derivative N-hydroxycytidine (NHC). The active form as NHC triphosphate targets viral RNA polymerase and has been shown to inhibit the replication of several RNA viruses including SARS-CoV-2.^4–6^ Significant clinical benefits have been demonstrated in a large phase 3 clinical trial for COVID-19 patients orally administered with molnupiravir.^7^ It is the first approved oral antiviral agent for treating COVID-19. However, the existing experimental and clinical data are based on the infection of the wild type (WT) or previous variants of SARS-CoV-2. Thus, this study aims to assess the effectiveness of molnupiravir on Omicron variant by using an isolate which was successfully cultured from an infected patient in the Netherlands. Interestingly, we observed that Omicron, as comparted to WT SARS-CoV-2, showed a reduced capability of propagation in human lung epithelial Calu-3 cells (Supplementary information, Fig. S1). In these cells inoculated with the virus and treated with molnupiravir, we found potent and dose-dependent inhibition of viral replication based on qRT-PCR quantification of intracellular viral genomic RNA (Fig. 1a). At clinically relevant concentration of 1 μM, molnupiravir already reduced Omicron viral RNA by 70.4% ± 12.5% (mean ± SD, *n* = 5; *P* = 0.0043). Complete inhibition was achieved at 10 μM and higher concentrations. The estimated half maximal inhibitory concentration (IC_50_) of molnupiravir was 1.965 μM to WT SARS-CoV-2 and 0.7556 μM to the Omicron variant. The dose response curves indicated that Omicron, as compared to WT SARS-CoV-2, appears to be somewhat more sensitive to the treatment in this model, especially at low concentrations ranging from 0.5 μM to 5 μM, and no cytotoxicity was observed (Fig. 1b). Consistently, viral titers quantified by a TCID50 assay confirmed potent inhibition of the production of infectious viruses by molnupiravir treatment (Fig. 1c). For example, treatment with 5 μM molnupiravir resulted in >900-fold decrease in infectious titers of Omicron, and production of infectious virus became undetectable by treatment of 20 μM molnupiravir (Fig. 1c). Immunostaining of viral nucleocapsid protein further confirmed the potent inhibition (Fig. 1d).

**Fig. 1:**
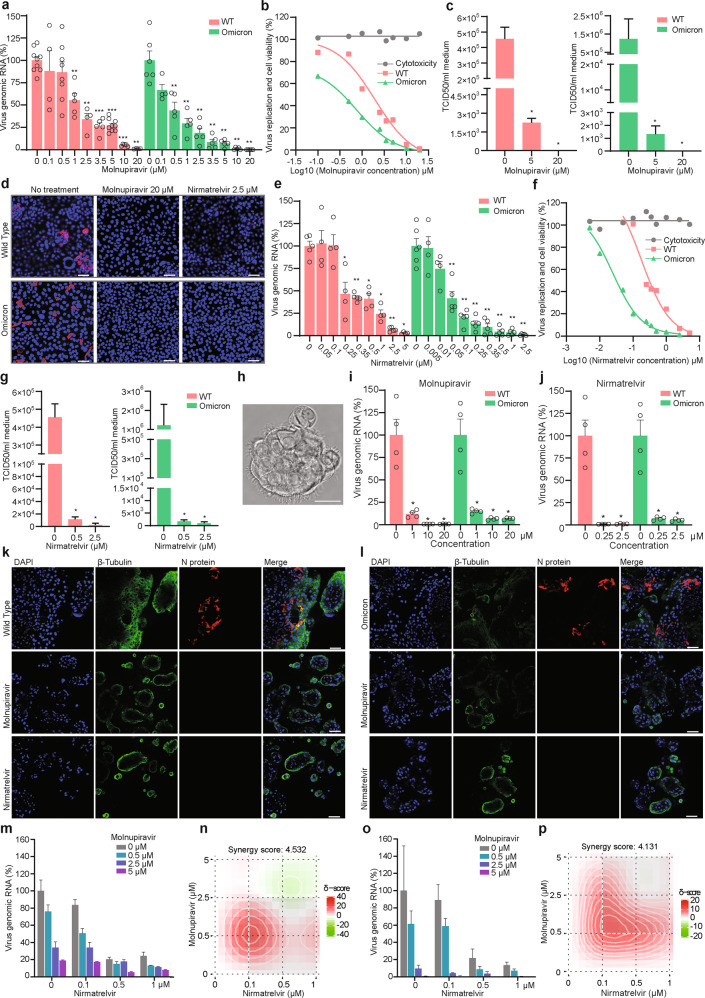
Antiviral activity of molnupiravir and nirmatrelvir against WT and Omicron SARS-CoV-2 infection in Calu-3 cells and human airway organoids. **a** The effects of molnupiravir on intracellular viral RNA levels of WT and Omicron SARS-CoV-2 in Calu-3 cells (*n* = 4–9). **b** Cell viability and virus replication curves in Calu-3 cells treated with molnupiravir (*n* = 4–9). **c** TCID_50_ assay quantifying titers of secreted infectious WT and Omicron SARS-CoV-2 virus particles at 48 h post treatment of molnupiravir (*n* = 4–5). **d** Immunofluorescence staining of nucleocapsid protein of SARS-CoV-2. Scale bar, 50 μm. **e** The effects of nirmatrelvir on intracellular viral RNA levels of WT and Omicron SARS-CoV-2 (*n* = 4–6). **f** Cell viability and virus replication curves by nirmatrelvir treatment (*n* = 4–8). **g** TCID_50_ assay quantifying titers of WT and Omicron SARS-CoV-2 at 48 h post treatment of nirmatrelvir (*n* = 4–5). **h** Bright field of hAOs at two weeks after differentiation towards proximal phenotype. Scale bar, 20 μm. **i**, **j** The effects of molnupiravir (**i**) (*n* = 4) and nirmatrelvir (**j**) (*n* = 4) on intracellular viral RNA levels of WT and Omicron SARS-CoV-2 in hAOs. **k**, **l** Immunofluorescence staining of SARS-CoV-2 nucleocapsid protein and ciliated cell marker β-tubulin in hAOs. Scale bar, 50 μm. **m** The antiviral effects of combining molnupiravir and nirmatrelvir in WT SARS-CoV-2-infected Calu-3 cells based on intracellular viral RNA levels (*n* = 3–4). **n** Synergy distribution of pairwise combination of molnupiravir and nirmatrelvir in WT SARS-CoV-2 infected Calu-3 cells (*n* = 3–4). **o** The antiviral effects of combining molnupiravir and nirmatrelvir in Omicron SARS-CoV-2 infected cells (*n* = 3–4). **p** Synergy distribution of pairwise combination of molnupiravir and nirmatrelvir in Omicron SARS-CoV-2-infected cells (*n* = 3–4). Data are presented as means ± SEM; *P < 0.05; **P < 0.01; ***P < 0.001.

On December 22, 2021, the U.S. Food and Drug Administration (FDA) has authorized the emergency use of PAXLOVID, another oral antiviral therapy for treating COVID-19. Nirmatrelvir is the key component of PAXLOVID, which inhibits the main protease of SARS-CoV-2.^8^ Results from a large phase 2/3 trial reported an 89% reduction in the risk of hospitalization or death for COVID-19 patients treated with PAXLOVID.^9^ Given that the effects of nirmatrelvir on the Omicron variant remain unknown, we treated Omicron-infected Calu-3 cells with a series of concentrations of nirmatrelvir. Potent and dose-dependent inhibition of intracellular viral genomic RNA was observed (Fig. 1e). A low concentration of 0.1 μM has already reduced Omicron viral RNA by 79.9% ± 8.3% (mean ± SD, *n* = 5; *P* = 0.0043). Complete inhibition was achieved at 2.5 μM and higher concentrations. The dose response curves calculated the IC_50_ of 0.1765 μM to WT and 0.02462 μM to Omicron SARS-CoV-2. These curves suggest that Omicron, compared to the parental WT SARS-CoV-2, is slightly more sensitive to nirmatrelvir in Calu-3 cell model, and no cytotoxicity was observed at the tested concentrations (Fig. 1f). Consistent with these results, quantification of viral titers (Fig. 1g) and staining of nucleocapsid protein (Fig. 1d) confirmed the potent inhibition. At concentration of 2.5 μM, nirmatrelvir treatment decreased infectious titers of Omicron by > 1200-fold (Fig. 1g). To mimic breakthrough infection in vaccinated individuals, we incubated Calu-3 cells with post COVID-19 vaccination serum and inoculated with WT or Omicron SARS-CoV-2. We found that WT SARS-CoV-2 failed to propagate in the presence of the serum, whereas Omicron remained capable of replicating albeit at a low level. Nevertheless, treatment of molnupiravir or nirmatrelvir effectively inhibited the residual replication (Supplementary information, Fig. S2).

The respiratory tract is lined by multi-ciliated cells, mucus-producing secretory cells and other columnar epithelium. Human airway organoids (hAOs) consisting of multiple cell lineages, including basal, ciliated, secretory and club cells, have been explored as advanced models for respiratory infections including influenza and SARS-CoV-2.^10,11^ Here, we employed differentiated hAOs to further validate the antiviral activity of molnupiravir and nirmatrelvir (Fig. 1h). In these hAOs inoculated with the virus and treated with both drugs, respectively, we found potent inhibition of viral replication based on intracellular viral RNA levels (Fig. 1i, j). For example, 10 μM molnupiravir and 0.25 μM nirmatrelvir treatments reduced >90% viral RNA level. Different from the observations in Calu-3 cell line (Fig. 1a, e), these two agents are marginally more potent in inhibiting the WT SARS-CoV-2 as compared to the Omicron variant in hAOs (Fig. 1i, j). Staining of viral nucleocapsid protein confirmed the effective inhibition of infection by treatment of molnupiravir (20 μM) and nirmatrelvir (2.5 μM) (Fig. 1k, l).

Although the results from the clinical trials of testing molnupiravir and nirmatrelvir are highly promising, there remains several obstacles with regard to their real-world implementation. Firstly, patients enrolled in these trials were treated during the early stage of the infection. Delivering antivirals to people within a few days of a positive diagnosis can be a challenge in practice. Secondly, despite of patient selection, the clinical outcomes remain suboptimal with monotherapy of molnupiravir or nirmatrelvir.^7,9^ An important lesson can be learned from treating hepatitis C virus (HCV) infection by direct-acting antivirals, which can cure nearly all the patients irrespective of the viral genotype and the patient condition. This transformative nature of anti-HCV therapy relies on the combination of protease and polymerase inhibitors.^12^ Therefore, we finally assessed the combination of molnupiravir and nirmatrelvir for treating SARS-CoV-2 infection in Calu-3 cells. We found synergistic antiviral activity of the combination against both WT and Omicron SARS-CoV-2 (Fig. 1m–p; Supplementary information, Fig. S3a–f). Interestingly, both molnupiravir and nirmatrelvir effectively inhibited viral replication of the Delta variant, but no synergistic effect was observed in the combination (Supplementary information, Fig. S3g–i). Drug combination has recently been proposed as a first line of defense against coronaviruses,^13^ and we expect that the combination of molnupiravir and nirmatrelvir is essential to enhance antiviral potency, limit toxicity, and avoid drug resistance in COVID-19 patients. Effective antiviral treatment, especially the rapid reduction of viral load, is expected to improve patient outcome but also limit virus transmission. When oral antiviral agents become widely accessible and affordable, the implementation in real world would represent a major innovation in combating the surge of the Omicron variant.

In summary, this study has demonstrated that molnupiravir and nirmatrelvir potently inhibited the infection of SARS-CoV-2 Omicron variant. Combination of molnupiravir and nirmatrelvir exerted synergistic antiviral activity. Of note, there are some subtle differences regarding the patterns of antiviral response among WT, Omicron and Delta variants, as well as between cell line and organoid models. Nevertheless, our findings support the use of molnupiravir and nirmatrelvir for treating Omicron-infected patients. We further call the initiation of clinical studies to evaluate the combination of molnupiravir and nirmatrelvir for treating COVID-19.

## Supplementary information

Supplementary Information

## References

[1] Callaway E (2021). Nature.

[2] Dejnirattisai, W. et al. *Lancet* **399**, 234–236 (2022). 10.1016/S0140-6736(21)02844-0 PMC8687667 34942101

[3] Kozlov, M. *Nature*10.1038/d41586-021-03829-0 (2021).

[4] Wang Y (2021). Virology.

[5] Wahl A (2021). Nature.

[6] Toots M (2019). Sci. Transl. Med.

[7] Jayk Bernal, A. et al. *N. Engl. J. Med.*10.1056/NEJMoa2116044 (2021).

[8] Owen DR (2021). Science.

[9] Mahase E (2021). BMJ.

[10] Zhou J (2018). Proc. Natl. Acad. Sci. USA.

[11] Lamers MM (2021). EMBO J.

[12] Stanciu C (2021). Expert Opin. Pharmacother..

[13] White JM (2021). mBio.

