# Supplementary material for: SARS-CoV-2 Omicron variant is highly sensitive to molnupiravir, nirmatrelvir, and the combination
Source: Cell Res. 2022 Jan 20;32(3):322–4. doi: 10.1038/s41422-022-00618-w (PMC8771185; doi:10.1038/s41422-022-00618-w)
Supplement: Supplementary file 1 — Supplementary Information [file 41422_2022_618_MOESM1_ESM.pdf]

Supplementary information to

SARS-CoV-2 Omicron variant is highly sensitive to molnupiravir, nirmatrelvir, and the combination

Supplementary Materials and Methods

Reagents and antibodies

Molnupiravir (MedChem Express, USA) and nirmatrelvir (MedChem Express, USA) were dissolved in dimethyl sulfoxide (DMSO, Sigma, Zwijndrecht, The Netherlands). Antibody against SARS-CoV-2 Nucleocapsid protein (mouse mAb) and β-Tubulin (rabbit mAb) were purchased from Invitrogen (Cat. MA5-29981) and Cell signaling (Cat. 2128), respectively.

Viruses and cell lines

Human lung epithelial cell line Calu-3 was cultured in advanced DMEM/F12 supplemented with 1% (vol/vol) GlutaMAXTM Supplement (Gibco, Grand island, USA), 10 mM HEPES, 100 IU/mL Penicillin and 100 mg/mL Streptomycin (Gibco, Grand Island, USA). Wild type (WT) SARS-CoV-2 (isolate BetaCoV/Munich/BavPat1/2020) was originally obtained from European Virus Archive Global. SARS-CoV-2 Delta and Omicron variants were isolated from infected patients at Department of Viroscience, Erasmus MC, The Netherlands. Cell lines were analyzed by genotyping and confirmed to be mycoplasma negative.

Human airway organoids

Adult lung tissues were obtained from residual, tumor-free, material obtained at lung resection surgery for lung cancer for culturing organoids. The Medical Ethical Committee of the Erasmus MC Rotterdam granted permission for this study (METC 2012-512). Human airway organoids (hAOs) were cultured in airway organoid expansion medium (AEM), based on advanced DMEM/F12 (Invitrogen), supplemented with 1% penicillin/streptomycin (Life Technologies), 1 M HEPES (Life Technologies), 200 mM Ultraglutamine (Life Technologies), 2% (vol/vol) of B27 (Gibco), 1.25 mM N-acetylcysteine (Sigma-Aldrich), 10 mM Nicotinamide (Sigma-Aldrich), 10% (vol/vol) of R-spondin-1

(conditioned medium), 10% (vol/vol) of Noggin (conditioned medium), 100 ng/ml FGF10 (Peprotech), 25 ng/ml FGF7 (Peprotech), 1 μM SB202190 (Tocris), 500 nM A83-01 (Tocris) and 10 μM Y27632 (Sigma-Aldrich). To differentiate airway organoids into proximal phenotype, culture medium was changed into complete base medium (CBM; Stemcell Pneumacult-ALI) supplemented with 10 uM DAPT (Tocris).

Virus inoculation and antiviral drug treatment

For Calu-3 cell line, cells were inoculated with SARS-CoV-2 virus particles (MOI = 0.02) for 1 hour at 37 °C. Cells were then washed by opti-MEM to remove unabsorbed viruses. Next, cells were supplemented with serial diluted antiviral drugs in opti-MEM and incubated at 37 °C with 5% CO_2. For hAOs, organoids were mechanically dissociated into small fragments and inoculated with SARS-CoV-2 virus particles (MOI = 0.1) for 1 hour at 37 °C. Organoids were then washed by PBS to thoroughly remove unabsorbed viruses. Finally, organoids were embedded in Matrigel, and maintained in CBM with serial diluted antiviral drugs at 37 °C with 5% CO_2. Cells and organoids were lysed to quantify virus genomic RNA expression after drug treatment for 48 h. For TCID 50 assay, supernatants were harvested at indicated time points and used to calculate TCID 50 in Calu-3 cell lines.

RNA isolation, cDNA synthesis and qRT-PCR

Total RNA was isolated using Macherey-Nagel NucleoSpin® RNA II kit (Bioke, Leiden, The Netherlands) and quantified using a Nanodrop ND-1000 (Wilmington, DE, USA). cDNA was synthesized by using a cDNA synthesis kit (TaKaRa Bio, Inc., Shiga, Japan). Real-time PCR reactions were performed with SYBR-Green-based real-time PCR (Applied Biosystems®, Austin, USA) on a StepOnePlusTM System (Thermo Fisher Scientific LifeSciences). Glyceraldehyde 3-phosphate dehydrogenase (GAPDH) gene was used as housekeeping gene. Relative gene expression of target gene was normalized to GAPDH using the formula $2^{-\Delta\Delta CT}$, ΔΔCT = ΔCTsample - ΔCTcontrol (ΔCT = CT [target gene] - CT[GAPDH]). Template control and reverse transcriptase control were included in all qRT-PCR experiments. The primers used are: GAPDH (F- GTCTCCTCTGACTTCAACAGCG; R-ACCACCCTGTTGCTGTAGCCAA), SARS-CoV-2 (F-CAATGGTTTAACAGGCACAGG; R-CTCAAGTGTCTGTGGATCACG).

MTT assay

Calu-3 cells were seeded into 96-well tissue culture plates (1×10^4cells/well), and then treated with the indicated compounds for 48 hours. Cells were incubated with 10 μL 5 mg/mL 3-(4,5-dimethyl-2-thiazolyl) -2,5-diphenyl-2H-tetrazolium bromide (MTT) for 3 hours, then replaced with 100 μL DMSO medium and incubated at 37°C for 30 minutes. The absorbance at 490 nm was recorded using a microplate absorbance reader (Bio-Rad, CA, USA).

TCID50 assay

Cell culture supernatants from virus infected Calu-3 cells were collected at indicated time points. Virus titer was quantified by using a 50% tissue culture infectious dose (TCID50) assay. Briefly, ten-fold dilutions of viruses were inoculated onto Calu-3 cells, grown in a 96-well tissue culture plate at 2,000 cells/well. The plate was incubated at 37 °C for 3-4 days, and each well was examined under a light microscope for cytopathic effect (CPE). The TCID50 value was calculated by using the Reed-Muench method.

Immunofluorescence assay

First, Calu-3 cells that were cultured in 8-well slides (Ibidi GmbH, Germany) or hAOs were fixed in 4% paraformaldehyde for 30 minutes. Subsequently, hAOs were added into the CytoSpin II Cytocentrifuge (Shandon Scientifi Ltd, Runcorn, England) and spun down into slides at 800 rpm for 5 min. The 8-well slides (Calu-3 cells) and slides containing organoids were then rinsed 3 times with PBS, followed by permeabilizing with PBS containing 0.2% (vol/vol) tritonX100 for 10 min. Then the slides were twice rinsed with PBS for 5 min, followed by incubation with blocking solution (5% donkey serum, 1% bovine serum albumin, 0.2% tritonX100 in PBS) at room temperature for 1 hour. Next, slides were incubated with primary antibody diluted in blocking solution at 4°C overnight. Primary antibodies used in this study are as follows: anti-β-Tubulin antibody (1:400, rabbit mAb; Cell signaling), anti-SARS-CoV-2 Nucleocapsid antibody (1:250, mouse mAb; Invitrogen). Slides were washed 3 times for 5 min each in PBS prior to 1 hour incubation with 1:1000 dilutions of the anti-mouse IgG (H+L, Alexa Fluor® 594), the anti-rabbit IgG (H+L, Alexa

Fluor® 488) secondary antibodies. Nuclei were stained with DAPI (4, 6-diamidino-2-phenylindole; Invitrogen).

Statistics

The statistical significance of differences between means was assessed with the Mann-Whitney test (GraphPad Prism; GraphPad Software Inc., La Jolla, CA). The threshold for statistical significance was defined as $P < 0.05$. Synergistic scores of drug combinations were analyzed by SynergyFinder 2.0[1].

Reference

1. Ianevski, A., Giri, A.K. & Aittokallio, T. SynergyFinder 2.0: visual analytics of multi-drug combination synergies. *Nucleic Acids Res* **48**, W488-W493 (2020).

Supplementary Figures

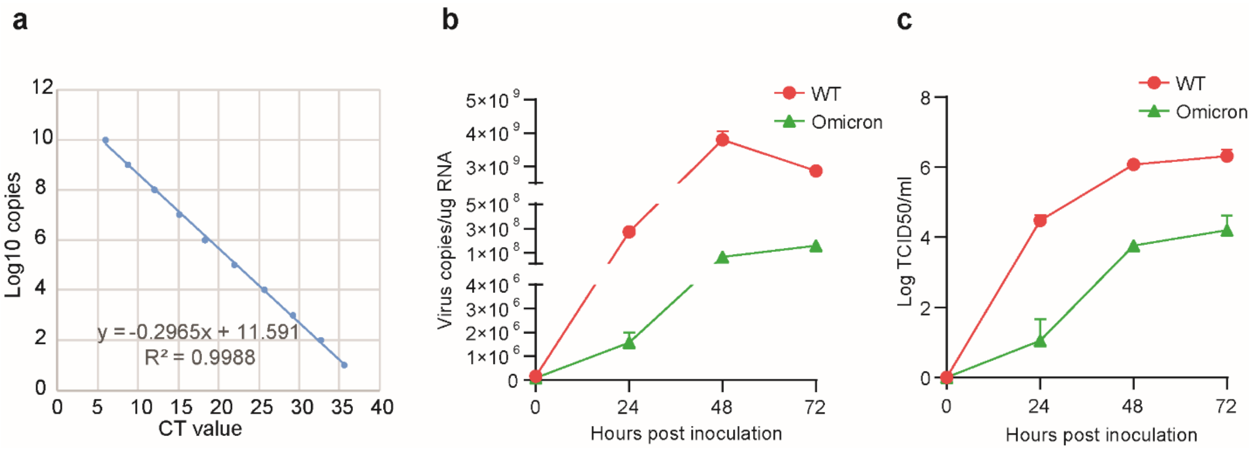

Fig. S1. The kinetics of wild type (WT) and Omicron SARS-CoV-2 infection in human lung epithelial Calu-3 cells. (a) Standard curve for quantification and calculation of SARS-CoV-2 genomic copy numbers. A plasmid containing partial SARS-CoV-2 genome harboring the target sequence was diluted from 10^{-1} to 10^{-10}, and amplified and quantified by qRT-PCR. Virus growth dynamics quantified by qRT-PCR of intracellular viral genomic RNA and was calculated as copy number (b), and TCID50 of produced infectious virus titers (c) in Calu-3 cells at 24, 48 and 72 hours post-inoculation. 0.01 MOI was used for inoculation, and 1 hour post virus inoculation was set as starting point of “0 hour”. Data presented as mean ± SEM; n = 4.

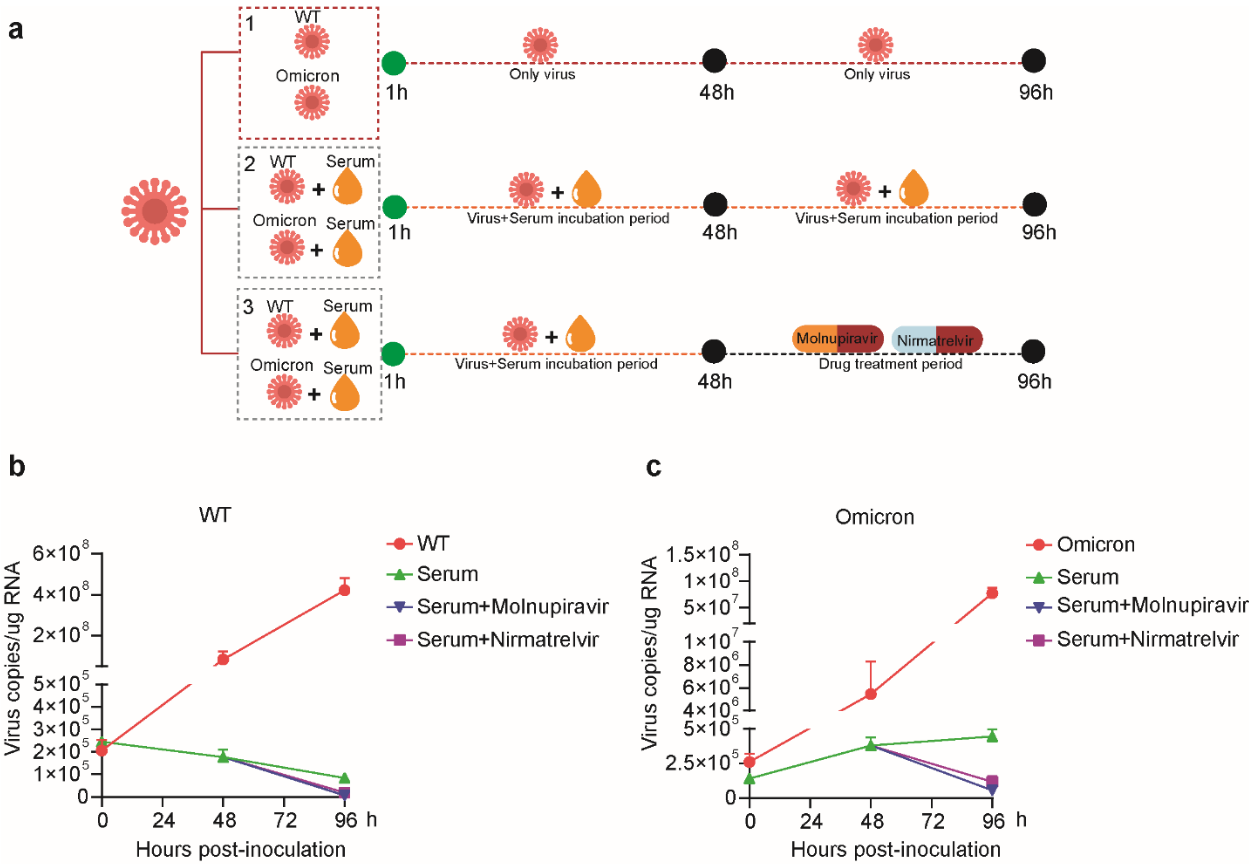

Fig. S2. Mimicking breakthrough infection and antiviral treatment. (a) Schematic illustration of experimental design. Group 1: Calu-3 cells were inoculated with wild type (WT) or Omicron SARS-CoV-2 respectively and incubated for 96 hours. Group 2: WT or Omicron viruses were firstly neutralized with post-vaccination (COVID-19 vaccine) serum for 1 hour, then inoculated Calu-3 cells and incubated for 96 hours. Group 3: WT or Omicron virus were firstly neutralized with post-vaccination serum, then inoculated Calu-3 cells and incubated for 48 hours, then culture supernatants were replaced with fresh medium supplemented with molnupiravir or nirmatrelvir. SARS-CoV-2 with 0.01 MOI was used to infect Calu-3 cells. Post-vaccination serum was pre-heated at 56 °C for 30 minutes and then diluted by 30-fold with opti-MEM. The replication dynamics of WT (b) and Omicron (c) SARS-CoV-2 was based on qRT-PCR quantification of genomic RNA and calculated as copy number. Data presented as mean ± SEM; n = 3.

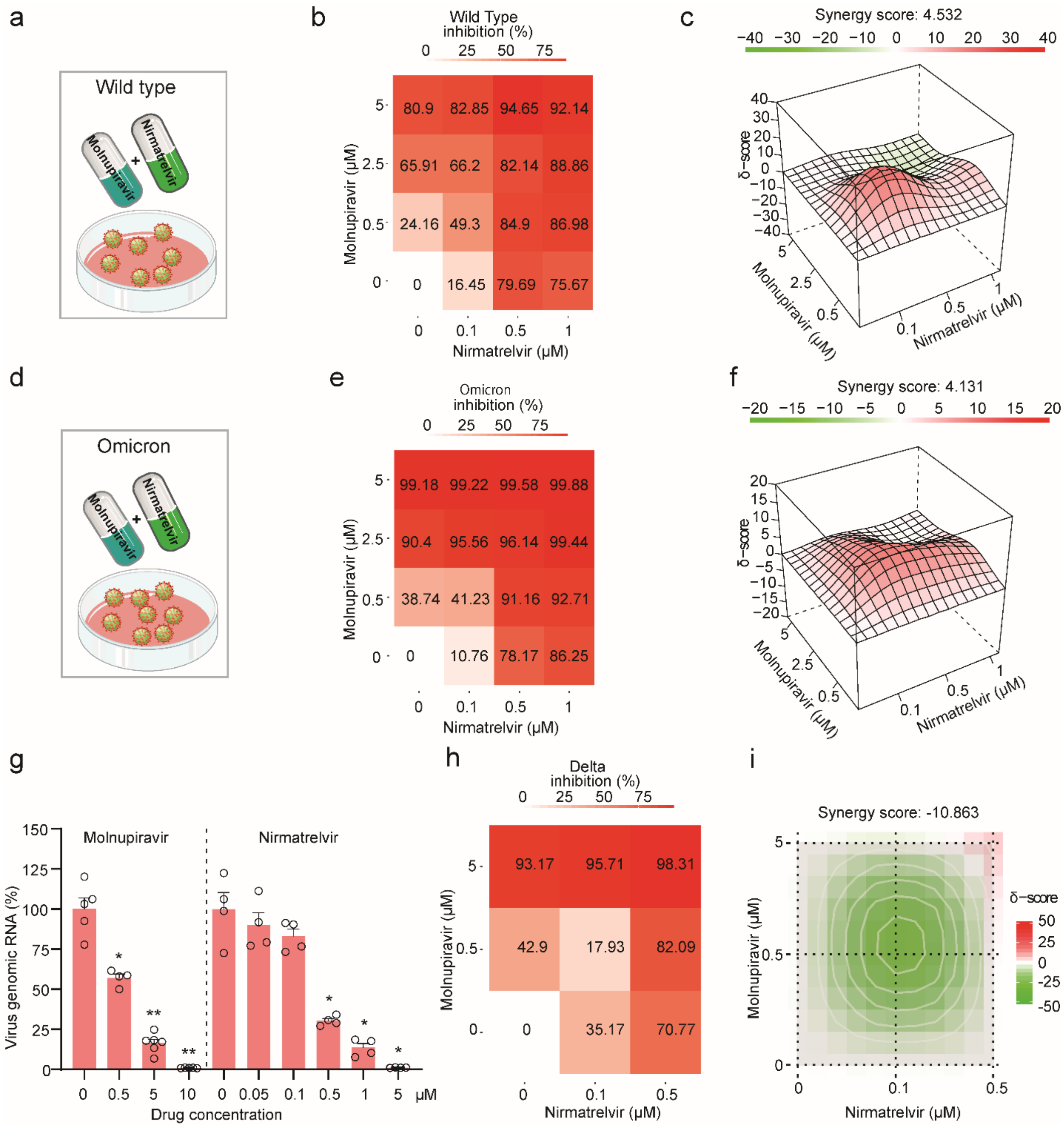

Fig. S3. The combination treatment of molnupiravir and nirmatrelvir. (a), Schematic representation of drug combination treatment in WT SARS-CoV-2 infected Calu-3 cells. (b, c), Dose-response matrix (b) and 3D synergy landscape (c) in treating wild type (WT) SARS-CoV-2 by combining molnupiravir and nirmatrelvir. (d), Schematic representation of drug combination treatment in Omicron SARS-CoV-2 infected Calu-3 cells. (e, f) Dose-response matrix (e) and 3D synergy landscape (f) in treating Omicron SARS-CoV-2 by combining molnupiravir and nirmatrelvir. (g) The effects of molnupiravir or nirmatrelvir on SARS-CoV-2 Delta variant. (h, i), Dose-response matrix (h) and Synergy distribution of pairwise combination (i) of molnupiravir and nirmatrelvir in treating Delta variant. Data presented as mean ± SEM; n = 4-6. *P<0.05; **P<0.01.
